# WE‐UNet: A Wavelet‐enhanced U‐Net framework for radiation dose reduction in chest radiography

**DOI:** 10.1002/acm2.70583

**Published:** 2026-05-13

**Authors:** Emil I. Cohen, Ufaq Khan, Benjamin Wallace, Arash R. Zandieh, Nariman Nezami, Ross W. Filice, David H. Field, William Poulett, Shazad Ashraf, Muhammad Bilal

**Affiliations:** ^1^ Department of Radiology MedStar Georgetown University Hospital Georgetown University Washington District of Columbia USA; ^2^ Department of Computer Vision Mohamed bin Zayed University of Artificial Intelligence Abu Dhabi UAE; ^3^ School of Computing and Digital Technology Birmingham City University Birmingham UK

**Keywords:** Deep learning, Low‐dose imaging, Radiation dose reduction, U‐Net, Wavelet

## Abstract

**Background:**

Reducing ionizing radiation exposure is a critical goal guided by the as‐low‐as‐reasonably‐achievable (ALARA) principle. Aggressively lowering radiation doses in radiography, however, amplifies image noise, compromising diagnostic quality.

**Purpose:**

To evaluate a hybrid Wavelet Enhanced‐UNet (WE‐UNet) model for denoising chest radiographs (CXRs), enabling substantial radiation dose reduction while maximizing diagnostic utility.

**Methods:**

A training dataset of 3000 images was created by simulating low‐dose conditions (70%–90% dose reduction) on CXRs from a public NIH dataset using Poisson‐Gaussian noise modeling. WE‐UNet was compared against four established architectures (DnCNN, REDNet, U‐Net, MWCNN) using quantitative metrics (MSE, MAE, PSNR, SSIM, edge preservation). Qualitative assessment was performed by two board‐certified radiologists in a blinded review.

**Results:**

Among five architectures, WE‐UNet achieved the highest SSIM (0.963 ± 0.007) and edge preservation (0.740 ± 0.116; *p < *0.001), while DnCNN showed comparable but not significantly better MSE and PSNR. Denoised images maintained PSNR of 35.23 ± 1.11 dB at 90% dose reduction. In blinded review, radiologist quality ratings for denoised images were statistically equivalent to original full‐dose acquisitions (*p *= 0.029, non‐significant after correction).

**Conclusions:**

WE‐UNet outperformed baseline models in structural preservation while maintaining image quality similar to standard‐dose acquisitions across dose reductions of 70%–90%, supporting its potential for significant radiation dose reduction.

## 1 INTRODUCTION

Chest radiographs (CXRs) are often the first‐line imaging test for numerous clinical conditions,^1^ with more than two billion examinations performed each year.^2^ Their popularity comes from three practical advantages, including speed (exposure takes milliseconds), accessibility (portable systems can be rolled to the bedside), and cost (a fraction of that for CT or MRI).^3^ However, all diagnostic radiographs expose the patient to ionizing radiation and its known stochastic and deterministic risks.^4^

Radiology departments therefore operate under the ALARA doctrine,^5^ which states “As Low As Reasonably Achievable” and guides clinicians to use the lowest radiation dose that still yields a diagnostic image.^6^ Digital detectors, automatic exposure control, and refined acquisition protocols have already reduced typical CXR doses to approximately 0.05 mSv (about the dose of a trans‐Atlantic flight).^7^ Although a single CXR delivers one of the lowest doses in diagnostic imaging, cumulative exposure becomes clinically relevant for patients requiring frequent serial examinations, such as those in intensive care or post‐operative monitoring. Dose reduction therefore, remains a priority, particularly for vulnerable populations such as pediatric patients and pregnant women, where radiosensitivity is elevated and the lifetime risk window is longest. Ionizing radiation also is used in other imaging modalities, including coronary angiography, fluoroscopy, and interventional radiology.^8^, ^9^, ^10^

Reducing ionizing radiation comes with its own set of challenges. Lower dose means that fewer X‐ray photons interact with the tissue and, subsequently, the detector, which inherently increases the noise in the resultant images. In simpler terms, when fewer photons are used to form an image, the picture becomes grainier, and important details can be obscured by this noise. This presents a dilemma, which means that while reducing the dose improves patient safety, it can potentially compromise the diagnostic quality of the image.

Traditional noise reduction methods, such as mean and Gaussian smoothing, were developed to address this issue.^11^ More advanced approaches, including wavelet‐based denoising and nonlocal means filtering, are also used.^12^ These methods distinguish noise and true anatomical details by analyzing image data in the frequency domain.^13^

In recent years, the field of deep learning has revolutionized many areas of image processing, including medical imaging.^14^, ^15^, ^16^ Convolutional neural networks (CNNs),^17^ in particular, have proven to be useful in extracting complex spatial features, making them suitable for a variety of tasks such as segmentation, super‐resolution, and denoising.^18^, ^19^, ^20^, ^21^ A wellknown deep learning model called U‐Net^22^ has become popular in the medical field thanks to its unique design and ability to address multiple challenges with small training datasets.

One of the key obstacles in applying deep learning to medical image denoising is the limited availability of high‐quality training data, including paired datasets containing low and standard‐dose images. For ethical and practical reasons, collecting large numbers of these paired images is challenging.^23^ Researchers simulate low‐dose conditions by adding realistic noise to standard‐dose images to overcome this issue.^24^, ^25^, ^26^, ^27^ By using these synthetic images, models can be trained effectively without the need for real images.^28^

Work in these areas has previously involved integrating traditional signal processing methods, such as wavelet‐based denoising,^29^ into deep learning frameworks. The advantage of using wavelets is that they allow the image to be split into different frequency components.

In doing so, noise usually appears as smaller, less significant coefficients, whereas important anatomical features appear as larger coefficients.^30^

This work presents and validates a hybrid approach that combines wavelet transforms with deep learning to enable acceptable CXR at radiation doses reduced by 70%–90%. Because the method operates on acquired images rather than acquisition hardware, it establishes a software‐based denoising framework transferable to higher‐dose modalities such as fluoroscopy and interventional procedures, where dose reduction would yield more substantial absolute benefits.

## 2 MATERIALS AND METHODS

### 2.1 Dataset and preprocessing

This study utilized a publicly available National Institutes of Health (NIH) CXR dataset,^31^ which contains over 100,000 anonymized frontal‐view CXRs. From this repository, 3000 frontal‐view (PA/AP) images were selected. The publicly available dataset provides processed images only; original acquisition parameters, detector specifications, and manufacturer information are not included in the released data. The dataset was randomly partitioned into training (80%, *n* = 2400) and validation (20%, *n* = 600) sets.

All selected images were resized to a uniform 512 × 512 pixel dimension to standardize the input format for our model. After resizing, each image was normalized to a pixel value range of [0, 1]. We also introduced data‐augmentation techniques, such as random cropping, which helped the network learn to adjust to variations in positioning.

A separate held‐out test set of 150 images was randomly sampled from the remaining approximately 97,000 images in the NIH dataset that were excluded from training and validation, ensuring full independence and no data leakage. Each test image was processed through all five deep learning architectures (WE‐UNet, DnCNN, REDNet, U‐Net, MWCNN) for quantitative comparison against original full‐dose references. For qualitative assessment, 46 images were randomly selected from the 150‐image test set. Three versions of each were generated (original full‐dose, simulated low‐dose at 70%–90% dose reduction, and WE‐UNet denoised), yielding 138 images.

Appropriate sample size for detecting medium effect sizes in radiologist qualitative image assessment across the three categories (original, denoised, noisy) was determined through power analysis. This analysis assumed a medium effect size (Cohen's *h* = 0.5), corresponding to approximately 20%–25% differences in classification accuracy between image types. With an alpha level of 0.05 and desired power of 90%, the analysis indicated that 40 images per category would be required, yielding a total minimum sample of 120 images. Two board‐certified radiologists (11 and 14 years of post‐fellowship experience) independently evaluated the same 138‐image set via a web‐based system, enabling assessment of inter‐rater reliability (link to site will be made available with publication).

### 2.2 Low‐dose simulation

Kroft et al. have previously demonstrated that CXRs acquired at up to 50% dose reduction remained diagnostically acceptable even without post‐processing.^32^ Therefore, a more aggressive reduction range of 70%–90% was simulated to ensure the study targeted a clinically meaningful scenario and also to build a model that can generalize to real‐world situations where multiple factors can produce different levels of image degradations even in the same patient.

Low‐dose image sets were simulated by applying established noising techniques to processed “for‐presentation” images provided by the NIH dataset.^33^, ^34^ It should be noted that these images have undergone post‐processing and do not preserve a direct linear relationship between pixel value and detector photon count. The Poisson‐Gaussian model has been validated to approximate the noise characteristics of detectors, accounting for both quantum mottle and electronic readout noise.^35^ A study by Lee et al in 2018 validated this model's accuracy for low‐dose X‐rays by demonstrating that the statistical noise distribution of images acquired from a physical chest phantom consistently adheres to Poisson‐Gaussian characteristics across multiple decomposition scales.^28^

Full‐dose images were converted into a format that represents photon counts. In this step, each image is normalized so that the brightest parts correspond to a maximum of 5000 photons. This conversion provides a clear and measurable baseline for the amount of radiation present in the image. The reduced dose was simulated by applying a random proportionate scaling factor to the photon counts, effectively reducing the number of photons by 70%–90%.

Next, Poisson noise was introduced, which is the type of noise that occurs naturally when there are fewer photons. This randomness in photon detection is a key characteristic of low‐dose images. To further enhance realism of the simulation, a small amount of Gaussian noise with a standard deviation of 0.01 was added. This additional noise represents the minor electronic fluctuations and sensor variations that are typically seen in digital radiography and have been previously validated.^35^, ^36^

### 2.3 Wavelet transform and WE‐UNet architecture

**FIGURE 1:**
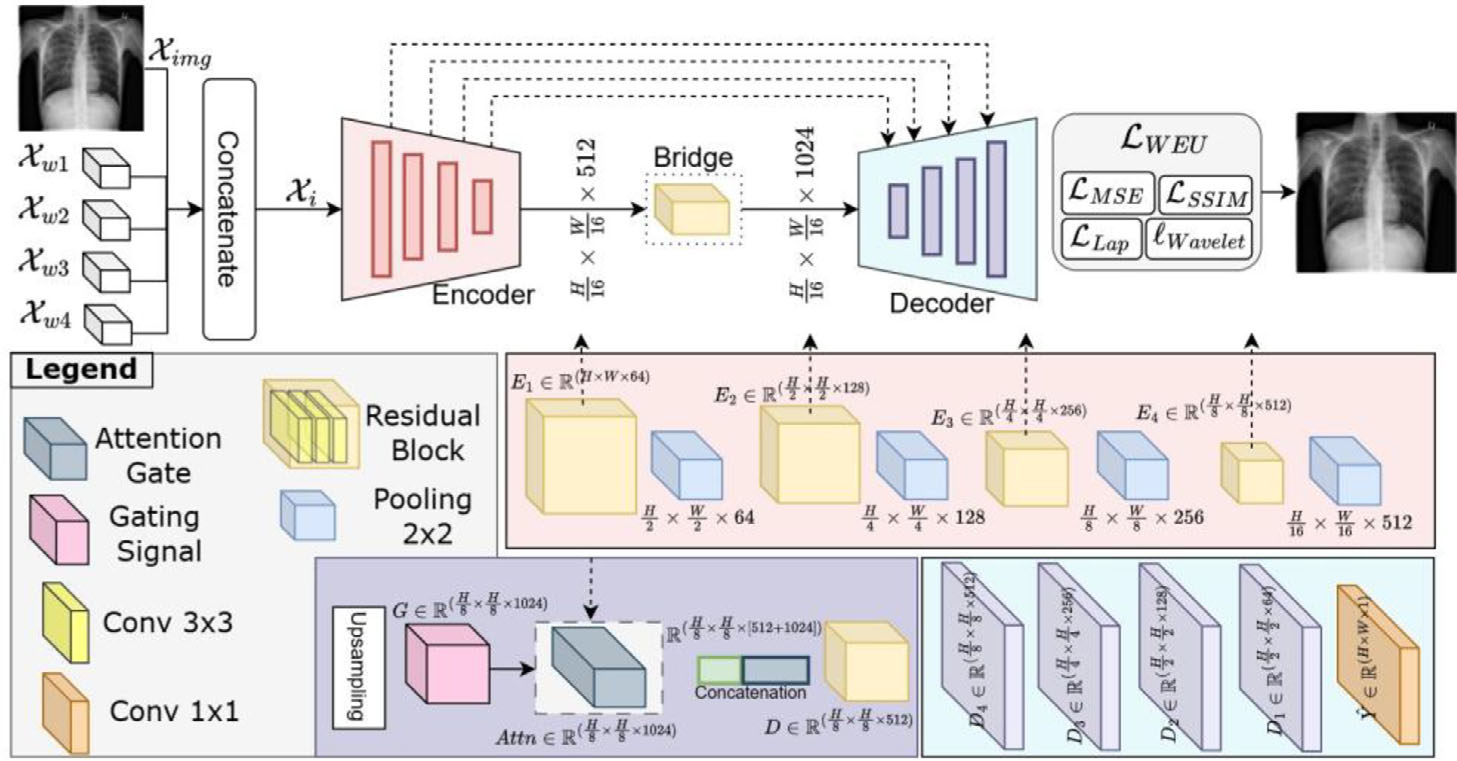
Five inputs, including the input image *X*_img_ and four wavelet–detail tensors {*X_w_*_1_, *X_w_*_2_, *X_w_*_3_, *X_w_*_4_} are concatenated to form *X_i_* ^∈^R*^H^* ^×^ *^W^* ^×13^. The encoder progressively down‐samples *X_i_* through four residual blocks (yellow), producing feature maps *E*_1_ −*E*_4_. A bridge forms the bottleneck. During decoding, each up‐sampled feature map acts as a gating signal (pink) for an attention gate (blue). The resulting tensor is concatenated and processed. A final 1 × 1 convolution outputs the denoised image.

A hybrid Wavelet‐Enhanced U‐Net (WE‐UNet) architecture was developed that combines traditional wavelet transforms with deep learning for enhanced denoising performance, adopted from similar networks published previously.^37^, ^38^, ^39^ The general architecture of the model employs a U‐Net backbone with residual blocks, attention gates, and skip connections to progressively encode features and reconstruct denoised images (Figure 1). Details of the complete architectural specifications are provided in Supplement A.

Four wavelet families were selected based on their complementary properties for the multi‐resolution decomposition: Daubechies (db5), Coiflet (coif1), Symlet (sym4), and Haar.^40^ Daubechies wavelets offer excellent time‐frequency localization with compact support, making them effective for capturing smooth intensity gradients in soft tissue regions.^41^ Symlet wavelets, a near‐symmetric modification of Daubechies, provide balanced noise reduction while preserving edge structures. Coiflet wavelets possess vanishing moments in both scaling and wavelet functions, which aids in preserving high‐contrast boundaries.^42^ Haar wavelets, despite their simplicity, excel at detecting sharp discontinuities and are computationally efficient.

Rather than selecting a single optimal wavelet, we adopted a multi‐wavelet input strategy that concatenates detail coefficients from four complementary wavelet families as explicit inputs to the U‐Net encoder. This approach differs from prior wavelet‐enhanced networks such as MWCNN,^43^ which employ wavelets primarily for architectural downsampling or learned pooling operations. By injecting multi‐resolution frequency information directly into the encoder pathway, the model learns to weight wavelet features according to their relevance for different structures. Comparative studies have shown that no single wavelet family consistently outperforms others across all medical imaging tasks^40^; the study model's integration strategy mitigates the bias associated with any fixed basis while jointly constraining spatial and frequency‐domain representations.

### 2.4 Training strategy

A systematic training approach in which the dataset was divided into smaller subsets of roughly 200 images each was used due to memory constraints. For each subset, 50 training epochs were performed using a batch size of four. After each epoch, we tested the model on a separate validation set comprising 20% of the images. If the validation loss showed improvement, the model weights were saved. The training was performed on NVIDIA A4000 GPU.

### 2.5 Evaluation metrics

#### 2.5.1 Quantitative evaluation

Quantitative evaluation employed five metrics computed between each denoised output and the corresponding original full‐dose reference: mean squared error (MSE), mean absolute error (MAE), peak signal‐to‐noise ratio (PSNR), structural similarity index (SSIM), and Edge Preservation Index. MSE and MAE assess overall intensity fidelity relevant to maintaining tissue contrast, PSNR quantifies signal quality relative to noise affecting the visibility of subtle findings, SSIM captures perceptual structural preservation of anatomical features, and the Edge Preservation Index evaluates retention of boundaries critical for identifying margins of pathology. The Edge Preservation Index was computed as the Pearson correlation coefficient between Laplacian‐filtered maps of the denoised and reference images. All metrics were computed after percentile‐based intensity normalization (1st–99th percentile) to ensure accurate comparison across different model outputs.^44^

#### 2.5.2 Qualitative evaluation

In addition to these objective measures, a qualitative evaluation was performed by two board‐certified radiologists. A website was setup (link available in supplement) in which the radiologist was shown one of three types of images at random(original, simulated noise, and denoised). The radiologist was then asked to rate the image on a 5‐point Likert scale and to label the image as either original, noisy or denoised.

### 2.6 Statistical analysis

For multi‐model comparisons, distributional assumptions were evaluated prior to statistical analysis. Shapiro–Wilk tests revealed significant departures from normality for all metrics across all models (all *p < *0.001). Therefore, the Friedman test (nonparametric repeated measures ANOVA) was used to assess whether significant differences existed among all five architectures for each metric. When significant (*p < *0.05), post‐hoc pairwise comparisons were performed using Wilcoxon signed‐rank tests. Bonferroni correction was applied across all 50 pairwise comparisons (10 model pairs × 5 metrics), yielding a corrected significance threshold of *α *= 0.001.

For qualitative radiologist assessments, one‐way ANOVA compared normalized quality ratings across the three image categories (original, noisy, denoised), followed by post‐hoc pairwise *t*‐tests with Bonferroni correction (3 comparisons, *α *= 0.0167). Inter‐rater reliability was assessed using weighted Cohen's *κ*.

All analyses were performed in Python 3.8 using SciPy (version 1.7.3) and NumPy (version 1.21.2).

### 2.7 Comparative analysis with baseline models

WE‐UNet performance was compared against four established deep learning architectures for image denoising. REDNet^45^ utilizes a symmetric encoder‐decoder structure with skip connections between corresponding convolutional and deconvolutional layers. The standard U‐Net^22^ architecture served as a direct comparison to isolate the contribution of wavelet enhancement in our proposed model. DnCNN^46^ employs residual learning with batch normalization to predict noise residuals directly, representing a foundational CNN‐based denoising approach. MWCNN^43^ integrates multi‐level discrete wavelet transforms into a CNN framework, using wavelet decomposition for downsampling rather than pooling operations.

All baseline models were trained using identical conditions. Evaluation was performed on the held‐out test set of 150 images using the same five metrics (MSE, MAE, PSNR, SSIM, Edge Preservation Index). Statistical significance between model pairs was assessed using Wilcoxon signed‐rank tests with Bonferroni correction (*α *= 0.001).

### 2.8 Dose‐level stratified analysis

To evaluate model robustness across varying levels of radiation reduction, a stratified analysis was performed at three discrete dose‐reduction levels: 70%, 80%, and 90%. For each level, 150 images from the held‐out test set were processed with the corresponding simulated noise intensity.

Again, all image quality metrics were computed by comparing both noisy and denoised images against the original full‐dose reference. Prior to metric calculation, all image pairs underwent percentile‐based intensity normalization.^44^

## 3 RESULTS

### 3.1 Quantitative evaluation and model comparison

**FIGURE 2:**
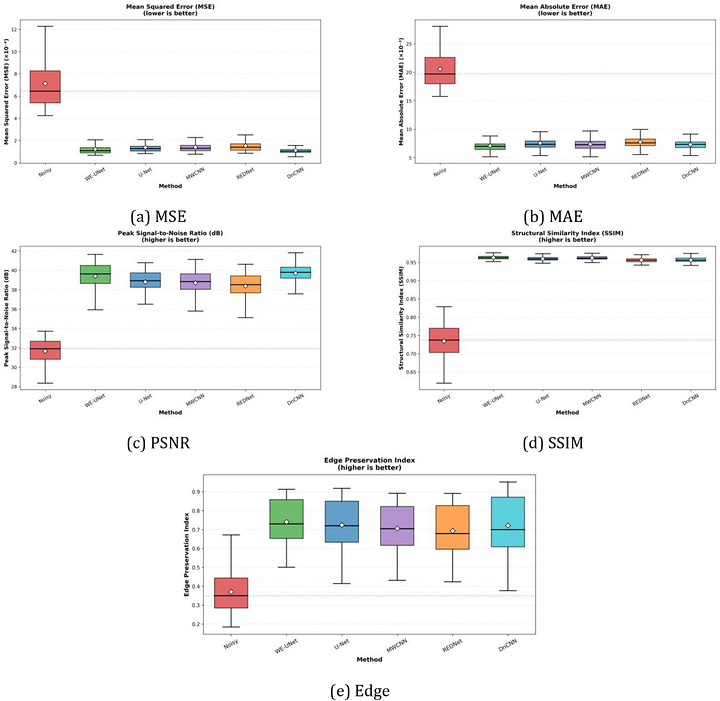
Box‐plot comparison of denoising performance for noisy baseline versus five deep learning models: (a) Mean squared error (MSE), (b) mean absolute error (MAE), (c) peak signal‐to‐noise ratio (PSNR), (d) structural similarity index (SSIM), and (e) Edge Preservation Index. Boxes span the interquartile range (IQR) with the median shown as a solid line; whiskers extend to 1.5× IQR. The horizontal dotted red line indicates the noisy baseline median (*n* = 150 images).

Table 1 summarizes the performance of all five deep learning architectures on the held‐out test set (*n* = 150). WE‐UNet achieved the highest SSIM (0.963 ± 0.007) and edge preservation (0.740 ± 0.116) among all models. For PSNR, WE‐UNet (39.39 ± 1.35 dB) performed comparably to DnCNN (39.69 ± 1.17 dB), with no statistically significant difference after Bonferroni correction (*p *= 0.025). Figure 2 presents box‐plot comparisons across all metrics.

**TABLE 1:** Model performance comparison (*n* = 150 images).

Model	MSE (×10^−4^)	MAE (×10^−3^)	PSNR (dB)	SSIM	Edge
WE‐UNet	1.21 ± 0.46	**7.11 **± **1.16**	39.39 ± 1.35	**0.963 **± **0.007**	**0.740 **± **0.116**
U‐Net	1.38 ± 0.51	7.58 ± 1.41	38.81 ± 1.29	0.960 ± 0.008	0.725 ± 0.126
MWCNN	1.41 ± 0.48	7.37 ± 1.16	38.70 ± 1.24	0.962 ± 0.007	0.706 ± 0.117
REDNet	1.53 ± 0.57	7.77 ± 1.29	38.38 ± 1.36	0.956 ± 0.008	0.694 ± 0.130
DnCNN	1.12 ± 0.35	7.30 ± 0.97	39.69 ± 1.17	0.957 ± 0.008	0.722 ± 0.157

*Note*: Values represent mean ± SD. Bold indicates best performance with statistical significance after Bonferroni correction (*p < *0.001).

**FIGURE 3:**
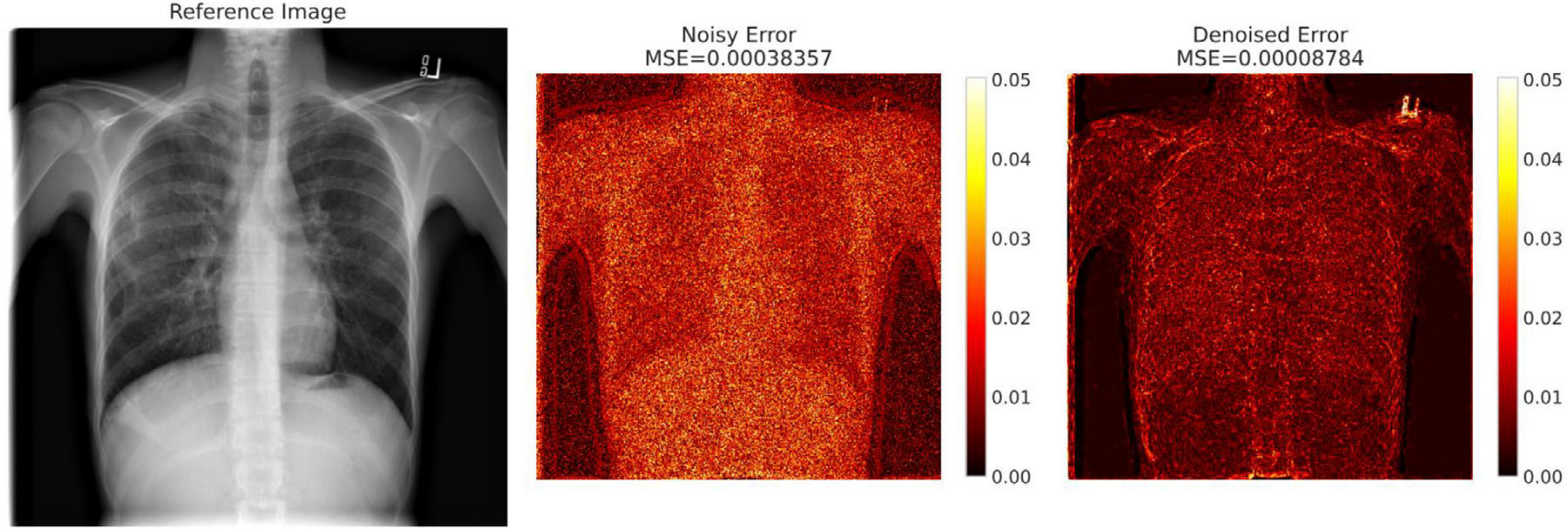
Sample chest X‐ray and corresponding absolute error maps for noisy versus denoised reconstructions.

**FIGURE 4:**
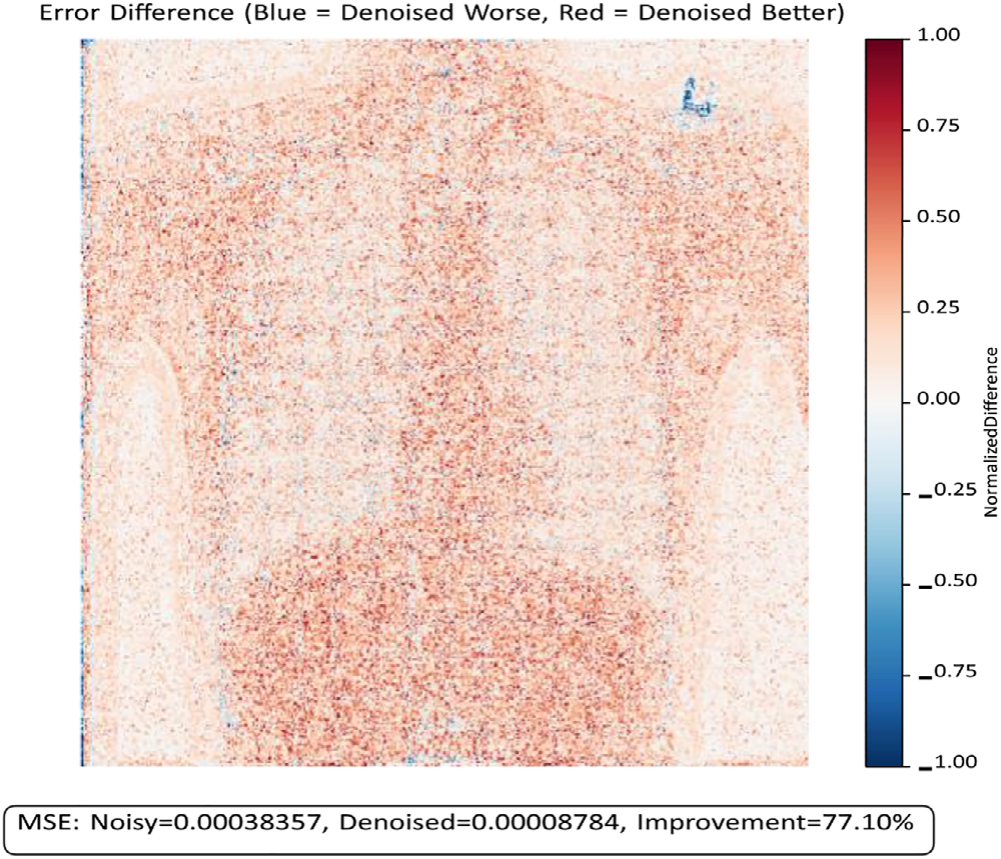
Spatial map of per‐pixel error difference between noisy and denoised reconstructions.

Figures 3 and 4 illustrate these quantitative improvements visually. The absolute error map (Figure 3) demonstrates a reduction in MSE from 3.8 × 10^−4^ (noisy) to 8.78 × 10^−5^ (denoised), a 77% improvement. The spatial difference map (Figure 4), derived from the same image shown in Figure 3, highlights regions where denoising improved (red) or worsened (blue) per‐pixel error relative to the original full‐dose reference image.

### 3.2 Qualitative analysis

**FIGURE 5:**
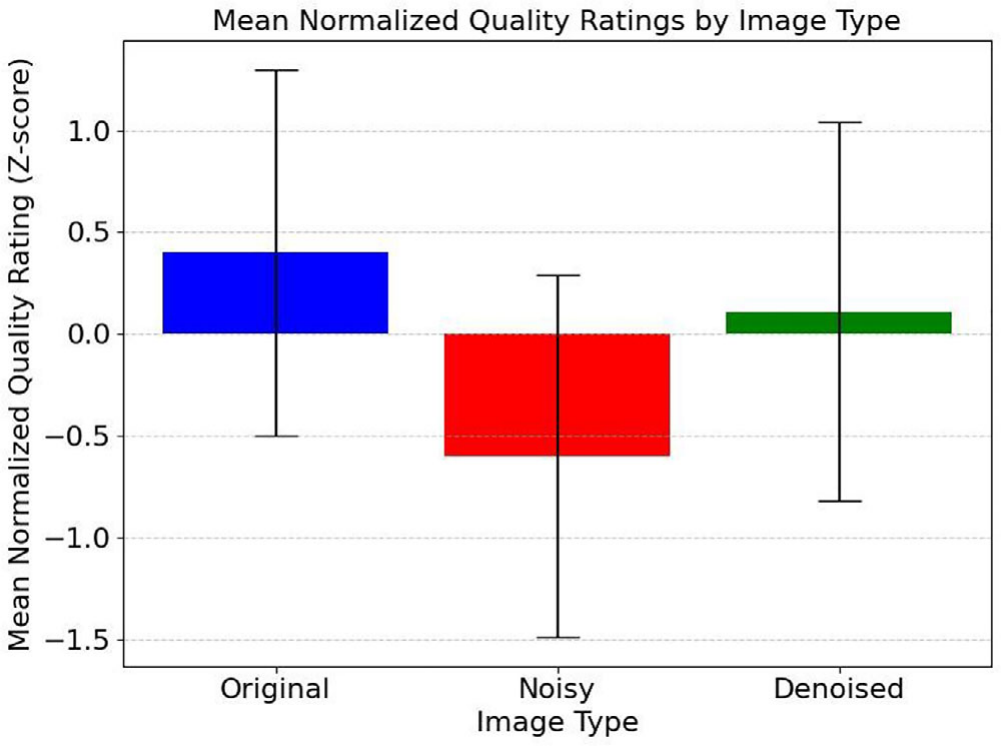
Mean normalized (z‐score) quality ratings for original, noisy, and denoised images.

**FIGURE 6:**
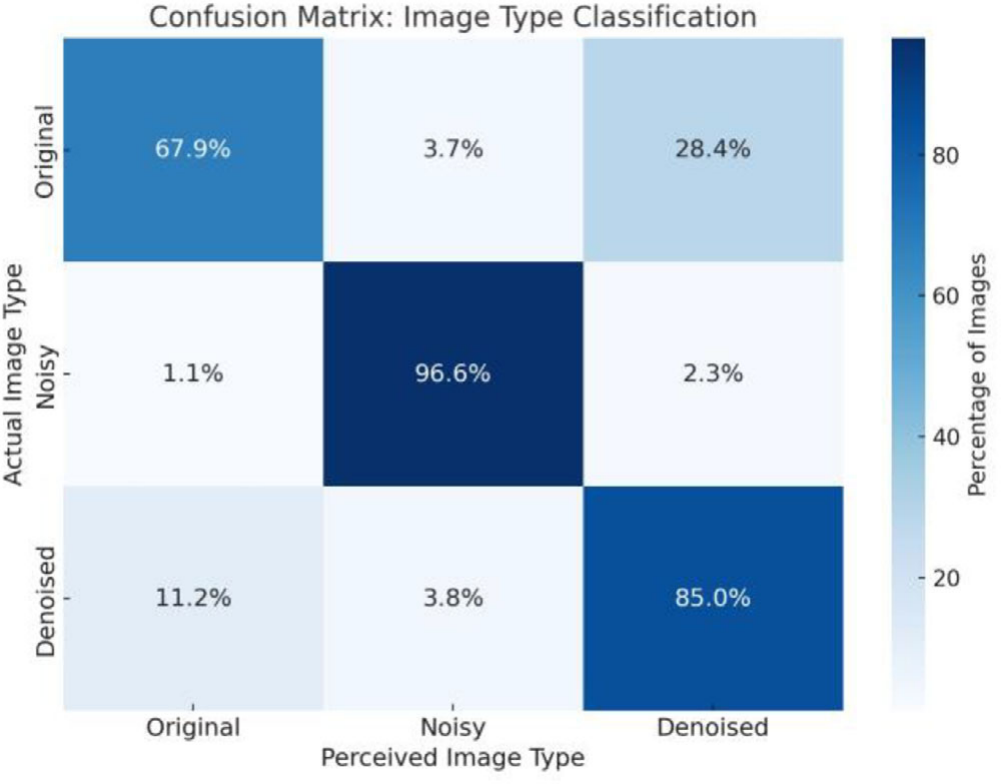
Confusion matrix for image‐type perception. Each row shows the percentage of original, noisy, and denoised images that were classified as “original,” “noisy,” or “denoised” (columns).

After normalization, original full‐dose images achieved the highest mean z‐score (0.40 ± 0.90), followed by denoised images (0.11 ± 0.89), with noisy images scoring lowest (−0.60 ± 0.93) as shown in Figure 5. One‐way ANOVA revealed significant differences between image types (*F *= 30.47, *p < *0.001). Post‐hoc pairwise comparisons with Bonferroni correction (Table 2) showed significant differences between original and noisy images (*p < *0.001, Cohen's *d *= 1.10) and between noisy and denoised images (*p < *0.001, Cohen's *d *= 0.78). Original images consistently scored highest in radiologist evaluation, but the difference was not significant after correction (*p *= 0.029). Notably, radiologists misclassified 28.4% of original images as denoised and 11.2% of denoised images as original, further supporting perceptual equivalence. Inter‐rater reliability was fair (weighted *κ *= 0.36). A confusion matrix is shown in Figure 6.

**TABLE 2:** Summary of ANOVA and post‐hoc test results.

Comparison	*p*‐value	Cohen's *d*	Significance
ANOVA	*<*0.001	–	Significant (*F *= 30.47)
Original vs. Noisy	*<*0.001	1.10	Significant
Noisy vs. Denoised	*<*0.001	0.78	Significant
Original vs. Denoised	0.029	–	Not Significant (*p <* 0.0167 required)

### 3.3 Performance across dose‐reduction levels

Table 3 presents WE‐UNet performance stratified by simulated dose reduction, with all metrics computed against the original full‐dose reference images using percentile‐based intensity normalization. At 70% dose reduction, denoised images achieved a mean PSNR of 39.39 ± 1.35 dB, representing an improvement of 7.73 ± 1.72 dB over the noisy inputs. SSIM reached 0.963 ± 0.007, a 31.6% improvement relative to the degraded images.

**TABLE 3:** WE‐UNet denoising performance stratified by simulated dose‐reduction level (*n* = 150 images per level). Values represent mean ± standard deviation.

Dose	Denoised	Denoised	Denoised	PSNR	Denoised	SSIM	Denoised
Reduction	MSE (×10^−4^)	MAE (×10^−3^)	PSNR (dB)	Improvement (dB)	SSIM	Improvement (%)	Edge
70%	1.21 ± 0.46	7.11 ± 1.16	39.39 ± 1.35	7.73 ± 1.72	0.963 ± 0.007	31.6 ± 8.9	0.740 ± 0.116
80%	1.97 ± 0.54	9.78 ± 1.67	37.21 ± 1.13	9.48 ± 1.55	0.946 ± 0.012	73.4 ± 13.6	0.702 ± 0.137
90%	3.10 ± 0.77	12.62 ± 2.14	35.23 ± 1.11	11.31 ± 1.56	0.923 ± 0.018	158.6 ± 29.8	0.646 ± 0.166

At 80% dose reduction, denoised PSNR decreased to 37.21 ± 1.13 dB while the improvement margin increased to 9.48 ± 1.55 dB, with SSIM of 0.946 ± 0.012 (73.4% improvement). At the most aggressive 90% dose reduction, denoised images maintained acceptable quality (PSNR: 35.23 ± 1.11 dB; SSIM: 0.923 ± 0.018), with the largest relative improvements observed (∆PSNR: +11.31 dB; ∆SSIM: +158.6%).

These results demonstrate an inverse relationship between input image quality and restoration benefit: as dose reduction increases and input images become more severely degraded, the relative improvement provided by WE‐UNet increases proportionally. Notably, even at 90% dose reduction, denoised images exceeded 35 dB PSNR.

## 4 DISCUSSION

The findings of this study underscore the effectiveness of WE‐UNet in denoising CXRs simulated at significantly lower radiation doses. Both quantitative and qualitative evaluations provide strong evidence that the proposed method reduces noise and improves image quality.

From a quantitative point of view, substantial drops in MSE and MAE confirm the precision of the model to match the reference intensity values. Furthermore, increased PSNR and SSIM scores underscore that the denoised images closely mimic the contrast and structural relationships of the original high‐dose scans. Among the five architectures evaluated, WE‐UNet achieved significantly higher SSIM and edge preservation than all baseline models, suggesting that the multi‐wavelet integration strategy enhances structural fidelity. DnCNN achieved comparable PSNR, indicating similar pixel‐wise reconstruction accuracy; however, its lower SSIM and edge preservation scores suggest that residual learning alone may sacrifice perceptual quality for intensity matching. These complementary performance profiles highlight a potential trade‐off between pixel‐level and structural metrics in denoising architectures.

**FIGURE 7:**
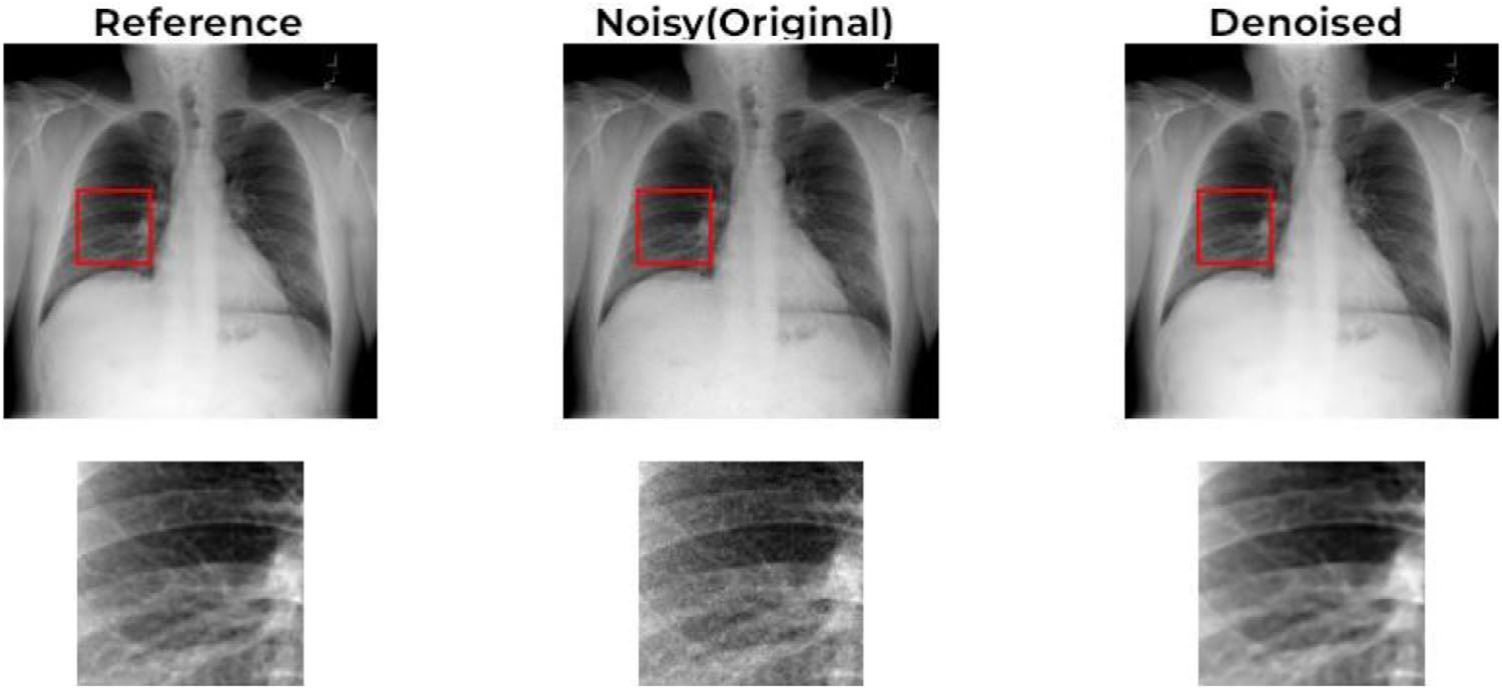
Sample improvement with zoom level.

Qualitatively, radiologist evaluation suggests that the denoised images align closely with the visual quality of the original high‐dose images (Figure 7).

Practical implications for emergency medicine and critical care deserve special consideration. Patients in these settings often require frequent imaging under challenging conditions, where motion artifacts and positioning limitations significantly compromise image quality. By enabling low‐dose acquisitions while maintaining diagnostic clarity, one can significantly benefit patients who face cumulative radiation exposure risks.^47^ Similarly, in pediatric patients, where radiation concerns are also justified, this technology could potentially enable routine dose reductions without sacrificing diagnostic confidence.

Global health applications represent another potential impact area. In resource limited settings where capital expenditures may be limited, older machines are used, and radiation protection measures may be less comprehensive.^48^ Software based dose reduction techniques could provide significant public health benefits to both patients and providers.

The dose‐level stratified analysis provides insight into model behavior under varying noise conditions. WE‐UNet demonstrated an inverse relationship between input image quality and restoration benefit, with greater improvements at higher noise levels (∆PSNR: +11.31 dB at 90% vs. +7.73 dB at 70% dose reduction). This suggests the multi‐wavelet decomposition effectively captures noise‐relevant features when degradation is most severe. However, absolute image quality decreases with dose reduction (PSNR: 39.39→35.23 dB), indicating inherent limits to restoration. Our results are comparable to recent deep learning approaches for low‐dose chest radiography; Jin et al. reported PSNR of 34–36 dB using CNN‐based denoising on chest X‐rays.^49^

The model has some limitations that warrant consideration. In particular, our training procedure relied on simulated low‐dose images rather than actual low‐dose acquisitions. The NIH dataset used provides processed images rather than raw detector data. As a result, the pixel values do not maintain a direct linear relationship with the incident photon counts. Applying the Poisson‐Gaussian noise model to these processed images, therefore, constitutes an empirical approximation, and the simulated noise distribution may not perfectly replicate that of true low‐dose acquisitions obtained from raw data.

Furthermore, although the combination of Poisson and Gaussian noise provides a reasonable and validated^35^ approximation of real imaging conditions, it may not capture every subtlety of clinical X‐ray detectors and scattering effects. It also does not fully capture the spatial noise correlations introduced by scintillator blurring or the effects of polyenergetic X‐ray spectra. Additionally, deep learning‐based denoising can introduce artifacts such as over‐smoothing of fine textures, hallucinated structures, or suppression of subtle pathological features that may be indistinguishable from noise.^50^ Furthermore, the dataset used was from a single institution (NIH) that has limitations in representing the variations present in numerous other settings where differences exist in both the patient population being examined and equipment being used. Imaging systems from different manufacturers employ distinct, anatomically aligned image processing pipelines that produce varying noise textures, contrast profiles, and edge characteristics.

Conducting prospective studies with real low‐dose datasets in various settings is necessary to further validate the model and assess whether denoising preserves diagnostically relevant features across diverse pathologies.

This study is also limited by the fair inter‐rater reliability (*κ *= 0.36), likely due to the lack of detailed scoring rubrics provided to radiologists subjectively rating the images. However, this is consistent with prior studies evaluating subjective image quality in dose reduction contexts, where similar inter‐observer variability (*κ *= 0.20–0.29) has been reported even with standardized criteria and trained readers.^51^ Furthermore, the qualitative evaluation used a general Likert scale rather than established anatomy‐specific image quality criteria such as those published by the European Commission. Future studies could incorporate such criteria to more directly assess the diagnostic utility of denoised images across specific anatomical structures. Finally, the relationship between clinical indication and acceptable image quality thresholds remains an important area for future investigation. Different diagnostic tasks (e.g., subtle nodule detection vs. assessment of line placement) impose varying demands on image fidelity, and the adequacy of denoised images may differ accordingly.

This study represents a proof‐of‐concept demonstration using 512×512 resolution images. Clinical CXRs have higher resolution, and scaling the model accordingly will require optimization of computational requirements and memory utilization. Real‐time processing may be necessary in certain applications, such as fluoroscopy, though for standard radiography, images can be processed prior to interpretation, thereby relaxing computational constraints. These implementation considerations should be addressed once a clinically scaled model is developed and validated. Furthermore, studies evaluating the model's impact on the depiction of specific pathologies and clinical diagnostic accuracy are essential before deployment to ensure denoising does not obscure or alter findings relevant to patient care.

## 5 CONCLUSION

Comparative analysis against four established deep learning architectures (DnCNN, REDNet, U‐Net, MWCNN) using rigorous statistical testing confirmed WE‐UNet's superior performance in structural similarity and edge preservation. Among all architectures tested, WE‐UNet achieved the highest SSIM (0.963 ± 0.007) and edge preservation (0.740 ± 0.116), while DnCNN showed comparable PSNR performance (39.69 ± 1.17 dB vs. 39.39 ± 1.35 dB; *p *= 0.025, not significant after Bonferroni correction). Dose‐stratified evaluation revealed an inverse relationship between input degradation and restoration benefit—the model provides the greatest improvement under severe noise conditions, though absolute image quality diminishes at higher dose reductions. Importantly, the PSNR from all denoised images, even those at 90% dose reduction, remained above the 35 dB threshold. Given that DnCNN achieved comparable PSNR while WE‐UNet excelled in structural preservation, ensemble approaches combining these complementary strengths warrant further exploration. In blinded review, radiologist ratings for denoised images did not differ significantly when compared to original images, supporting perceptual equivalence to standard‐dose quality. These results support WE‐UNet as a promising foundation for dose‐reduction denoising pipelines pending further validation.

These findings must be interpreted within methodological constraints: reliance on simulated rather than clinical low‐dose acquisitions, evaluation limited to a single‐institution dataset, fair inter‐rater agreement in qualitative assessment, and proof‐of‐concept implementation at 512 × 512 resolution requiring significantly more optimization and validation before clinical testing across a variety of pathologies ranging from subtle lung nodules to more prominent tissue characteristic differences noted in shadows of overlying structures.

The development of denoising models must be validated through prospective studies with actual low‐dose acquisitions in multi‐institutional testing. Another avenue that holds promise is application to other radiographic modalities that naturally use low‐dose imaging, such as fluoroscopy in diagnostic and interventional fields.

## AUTHOR CONTRIBUTIONS

**Emil I. Cohen**: Conceptualization; methodology; formal analysis; investigation; writing – review & editing; visualization; validation; resources. **Ufaq Khan**: Investigation; writing – original draft; writing – review & editing; visualization. **Benjamin Wallace**: Investigation; writing – review & editing; visualization; supervision. **Arash R. Zandieh**: Writing – review & editing; validation. **Nariman Nezami**: Investigation; writing – review & editing; visualization; validation; resources. **Ross W. Filice**: Conceptualization; methodology; investigation; writing – review & editing. **David H. Field**: Writing – review & editing; validation. **William Poulett**: Methodology; writing & editing; supervision. **Shazad Ashraf**: Methodology; software; formal analysis; investigation; writing – review & editing; supervision. **Muhammad Bilal**: Conceptualization; methodology; software; formal analysis; investigation; writing – original draft; writing – review & editing; visualization; supervision; validation.

## CONFLICT OF INTEREST STATEMENT

The authors declare no conflicts of interest related to this work.

## Supporting information



Supporting Information

## Data Availability

This study utilized the National Institutes of Health (NIH) chest radiograph dataset, available at: https://www.kaggle.com/datasets/nih‐chest‐xrays/data

## References

[1] Speets AM , van der Graaf Y , Hoes AW , et al. Chest radiography in general practice: indications, diagnostic yield and consequences for patient management. Br J Gen Pract. 2006;56(529):574‐578. 16882374 PMC1874520

[2] Akhter Y , Singh R , Vatsa M . Ai‐based radiodiagnosis using chest x‐rays: a review. Front Big Data. 2023;6:1120989. 37091458 10.3389/fdata.2023.1120989 PMC10116151

[3] Ou X , Chen X , Xu X , et al. Recent development in x‐ray imaging technology: future and challenges. Research. 2021. doi:10.34133/2021/9892152 10.34133/2021/9892152 PMC8724686 35028585

[4] Linet MS , Slovis TL , Miller DL , et al. Cancer risks associated with external radiation from diagnostic imaging procedures. CA Cancer J Clin. 2012;62(2):75‐100. 22307864 10.3322/caac.21132 PMC3548988

[5] Hansson SO . Alara: what is reasonably achievable?. Radioactivity in the Environment. Elsevier; 2013:143‐155.

[6] Oakley PA , Harrison DE . Death of the alara radiation protection principle as used in the medical sector. Dose Response. 2020;18(2):1559325820921641. doi:10.1177/1559325820921641 32425724 10.1177/1559325820921641 PMC7218317

[7] Arslan M , Haider A , Khurshid M , et al. From pixels to pathology: employing computer vision to decode chest diseases in medical images. Cureus. 2023;15(9):e45546. 37868395 10.7759/cureus.45587 PMC10587792

[8] Çimen S , Gooya A , Grass M , Frangi AF . Reconstruction of coronary arteries from x‐ray angiography: a review. Med Image Anal. 2016;32:46‐68. 27054277 10.1016/j.media.2016.02.007

[9] Lopez PD . Fluoroscopy history, evolution, and technological advancements: a narrative review. J Med Imaging Radiat Sci. 2024. doi:10.1016/j.jmir.2024.02.017 10.1016/j.jmir.2024.02.017 38565470

[10] Brock KK , Chen SR , Sheth RA , Siewerdsen JH . Imaging in interventional radiology: 2043 and beyond. Radiology. 2023;308(1):e230146. doi:10.1148/radiol.230146 37462500 10.1148/radiol.230146 PMC10374939

[11] Thanh DNH , Prasath S , Hieu LM . A review on ct and x‐ray images denoising methods. Informatica. 2019;43(2), 151‐159. doi:10.31449/inf.v43i2.2179

[12] Liu C , Zhang L . A novel denoising algorithm based on wavelet and non‐local moment mean filtering. Electronics. 2023;12(6):1461. doi:10.3390/electronics12061461

[13] Singh K , Kaur RS , Singh C . Comparative performance analysis of various wavelet and nonlocal means based approaches for image denoising. Optik. 2017;131:423‐437. doi:10.1016/j.ijleo.2016.11.055

[14] Li M , Jiang Y , Zhang Y , Zhu H . Medical image analysis using deep learning algorithms. Front Public Health. 2023;11:1273253. doi:10.3389/fpubh.2023.1273253 38026291 10.3389/fpubh.2023.1273253 PMC10662291

[15] Archana R , Eliahim Jeevaraj PS . Deep learning models for digital image processing: a review. Artif Intell Rev. 2024;57(1):11. doi:10.1007/s10462‐023‐10631‐z

[16] O'Mahony N , Campbell S , Carvalho A , et al. Deep learning vs. traditional computer vision. In: Advances in Computer Vision: Proceedings of the 2019 Computer Vision Conference (CVC) , Vol. 11, pages 128‐144. Springer, 2020.

[17] O'Shea K , Nash R. An introduction to convolutional neural networks. *arXiv*. Preprint posted online November 26, 2015. arXiv:1511.08458.

[18] Yuan F , Zhang Z , Fang Z . An effective CNN and transformer complementary network for medical image segmentation. Pattern Recognit. 2023;141:109228.

[19] Xing W , Egiazarian K. End‐to‐end learning for joint image demosaicing, denoising and super‐resolution. In: Proceedings of the IEEE/CVF Conference On Computer Vision and Pattern Recognition . 2021:3507‐3516.

[20] Zhang Q , Xiao J , Tian C , Lin CW , Zhang S . A robust deformed convolutional neural network (CNN) for image denoising. CAAI Trans Intell Technol. 2023;8(2):331‐342. doi:10.1049/cit2.12110

[21] Tian C , Zheng M , Zuo W , et al. Multi‐stage image denoising with the wavelet transform. Pattern Recognit. 2023;134. doi:10.1016/j.patcog.2022.109050

[22] Ronneberger O , Fischer P , Brox T . U‐net: convolutional networks for biomedical image segmentation. In: Medical Image Computing and Computer‐Assisted Intervention—MICCAI 2015: 18th International Conference; October 5‐9, 2015; Munich, Germany. Cham, Switzerland: Springer; 2015:234‐241.

[23] Leuschner J , Schmidt M , Otero Baguer D , Maass P . Lodopab‐ct, a benchmark dataset for low‐dose computed tomography reconstruction. Sci Data. 2021;8:109. 33863917 10.1038/s41597-021-00893-z PMC8052416

[24] Elhamiasl M , Nuyts J . Low‐dose x‐ray CT simulation from an available higher‐dose scan. Phys Med Biol. 2020;65(13):135010. doi:10.1088/1361‐6560/ab8953 32294635 10.1088/1361-6560/ab8953

[25] David‐Olawade AC , Olawade DB , Vanderbloemen L , et al. Ai‐driven advances in lowdose imaging and enhancement—a review. Diagnostics. 2025;15(6):689. 40150031 10.3390/diagnostics15060689 PMC11941271

[26] Hasinoff SW . Photon, poisson noise. In: Computer Vision: A Reference Guide. Springer; 2021:980‐982. doi:10.1007/978‐3‐030‐63416‐2

[27] Russo F . A method for estimation and filtering of gaussian noise in images. IEEE Trans Instrum Meas. 2003;52(4):1148‐1154. doi:10.1109/TIM.2003.815989

[28] Lee S , Lee M , Kang MG . Poisson‐gaussian noise analysis and estimation for low‐dose x‐ray images in the NSCT domain. Sensors. 2018;18(4):1019. doi:10.3390/s18041019 29596335 10.3390/s18041019 PMC5948630

[29] Halidou A , Mohamadou Y , Ari AAA , Zacko EJG . Review of wavelet denoising algorithms. Multimed Tools Appl. 2023;82(27):41539‐41569. doi:10.1007/s11042‐023‐15127‐0

[30] Ouahabi A . A review of wavelet denoising in medical imaging. In: 2013 8th International Workshop on Systems, Signal Processing and Their Applications (WoSSPA). IEEE; 2013:19‐26. doi:10.1109/WoSSPA.2013.6602330

[31] Wang X , Peng Y , Lu L , et al. Chestx‐ray8: hospital‐scale chest x‐ray database and benchmarks on weakly‐supervised classification and localization of common thorax diseases. In: Proceedings of the IEEE Conference on Computer Vision and Pattern Recognition . 2017:2097‐2106.

[32] Kroft LJ , Veldkamp WJ , Mertens BJ , van Delft JP , Geleijns J . Dose Reduction in Digital Chest Radiography and Perceived Image Quality. British Journal of Radiology. 2007;80(960):984‐988. 17940130 10.1259/bjr/80232832

[33] Veldkamp WJH , Kroft LJM , Delft JP , Geleijns J . A technique for simulating the effect of dose reduction on image quality in digital chest radiography. J Digit Imaging. 2009;22(2):114‐125. doi:10.1007/s10278‐008‐9104‐5 18259814 10.1007/s10278-008-9104-5 PMC3043684

[34] Tanaka R , Ichikawa K , Matsubara K , Kawashima H . Review of a simple noise simulation technique in digital radiography. Radiol Phys Technol. 2012;5(2):178‐185. doi:10.1007/s12194‐012‐0152‐7 22532067 10.1007/s12194-012-0152-7

[35] Foi A , Trimeche M , Katkovnik V , Egiazarian K . Practical poissonian‐gaussian noise modeling and fitting for single‐image raw‐data. IEEE Trans Image Process. 2008;17(10):1737‐1754. doi:10.1109/TIP.2008.2001399 18784024 10.1109/TIP.2008.2001399

[36] Eckert D , Seifert S , Pöppl S , et al. Guidance to noise simulation in x‐ray imaging. In: Maier A , Deserno T M , Handels H , Maier‐Hein K , Palm C , Tolxdorff T , eds. Bildverarbeitung für die Medizin 2024 (BVM 2024). Springer Vieweg; 2024:184‐189.

[37] Liu P , Zhang H , Lian W , Zuo W . Multi‐level wavelet convolutional neural networks. IEEE Access. 2019;7:74973‐74985. doi:10.1109/ACCESS.2019.2921451

[38] Zhao Y , Wang S , Zhang Y , Qiao S , Zhang M . Wranet: wavelet integrated residual attention u‐net network for medical image segmentation. Published online June 2023:1‐13. 10.1007/s40747-023-01119-y PMC10248349 37361970

[39] Kim W , Lee J , Kang M , Kim JS , Choi JH . Wavelet subband‐specific learning for low‐dose computed tomography denoising. PLoS One. 2022;17(9):e0274308. doi:10.1371/journal.pone.0274308 36084002 10.1371/journal.pone.0274308 PMC9462582

[40] Stanke L , Kubicek J , Vilimek D , et al. Towards to Optimal Wavelet Denoising Scheme—A Novel Spatial and Volumetric Mapping of Wavelet‐Based Biomedical Data Smoothing. Sensors. 2020;20(18):5301. doi:10.3390/s20185301 32947977 PMC7571247

[41] Daubechies I . Ten Lectures on Wavelets. Society for Industrial and Applied Mathematics (SIAM); 1992. doi:10.1137/1.9781611970104

[42] Kother MS , Arumuga Perumal S , Mohamed Sathik M . Image denoising using discrete wavelet transform. Int J Comput Sci Netw Secur. 2008;8(1):213‐216.

[43] Liu P , Zhang H , Zhang K , Lin L , Zuo W . Multi‐level wavelet‐CNN for image restoration. In: Proceedings of the IEEE Conference on Computer Vision and Pattern Recognition Workshops . 2018:773‐782.

[44] Nyu´l LG , Udupa JK . On standardizing the MR image intensity scale. Magn Reson Med. 1999;42(6):1072‐1081. doi:10.1002/(SICI)1522‐2594(199912)42:6%3c1072::AID‐MRM11%3e3.0.CO;2‐M 10571928 10.1002/(sici)1522-2594(199912)42:6<1072::aid-mrm11>3.0.co;2-m

[45] Mao X , Shen C , Yang YB . Image restoration using very deep convolutional encoder‐decoder networks with symmetric skip connections. In: Advances in Neural Information Processing Systems. 2016:2802‐2810.

[46] Zhang K , Zuo W , Chen Y , Meng D , Zhang L . Beyond a Gaussian denoiser: residual learning of deep CNN for image denoising. IEEE Trans Image Process. 2017;26(7):3142‐3155. doi:10.1109/TIP.2017.2662206 28166495 10.1109/TIP.2017.2662206

[47] McCollough CH , Leng S , Yu L , Fletcher JG . Low‐dose CT for the detection and classification of metastatic liver lesions: results of the 2016 low dose CT grand challenge. Med Phys. 2017;44(10):e339‐e352. doi:10.1002/mp.12345 29027235 10.1002/mp.12345 PMC5656004

[48] Mollura DJ , Mazal J , Everton KL . White Paper Report of the 2012 RAD‐AID Conference on International Radiology for Developing Countries: Planning the Implementation of Global Radiology. Journal of the American College of Radiology. 2013;10(8):618‐624. 23583085 10.1016/j.jacr.2013.01.019

[49] Kun J , Chen X , Lan Z , Wu J . Chest X‐ray image denoising method based on deep convolution neural network. IET Image Process. 2019;13(11):1970.

[50] Antun V , Renna F , Poon C , Adcock B , Hansen AC . On instabilities of deep learning in image reconstruction and the potential costs of AI. Proc Natl Acad Sci USA. 2020;117(48):30088‐30095. doi:10.1073/pnas.1907377117 32393633 10.1073/pnas.1907377117 PMC7720232

[51] Kataria B , Nilsson Althén J , Smedby O , et al. Assessment of image quality in abdominal CT: potential dose reduction with model‐based iterative reconstruction. Eur Radiol. 2018;28(6):2464. doi:10.1007/s00330‐017‐5113‐4 29368163 10.1007/s00330-017-5113-4 PMC5938296

