# Supplementary material for: WE‐UNet: A Wavelet‐enhanced U‐Net framework for radiation dose reduction in chest radiography
Source: J Appl Clin Med Phys. 2026 May 13;27(5):e70583. doi: 10.1002/acm2.70583 (PMC13172257; doi:10.1002/acm2.70583)
Supplement: Supplementary file 1 — Supporting Information [file ACM2-27-e70583-s001.pdf]

Supplementary Methods: WE-UNet

Supplementary Methods

0.1 Wavelet Transform and Image Decomposition

The Wavelet-UNet (WE-UNet) model incorporates four wavelet families[1]: *Daubechies* 5 (db5), *Coiflet* 1 (coif1), *Symlet* 4 (sym4), and *Haar*. For computational efficiency, GPU-accelerated separable convolutions were used to perform the wavelet transforms. Each wavelet produces three high-frequency detail maps (horizontal, vertical, diagonal). These maps were concatenated into a multichannel tensor, yielding 12 additional channels. Combined with the original grayscale input, this produced a 13-channel input tensor for the model.

0.2 WE-UNet Architecture

A modified U-Net[2] architecture was developed and named Wavelet-Enhanced U-Net (WE-UNet). The network accepts five input tensors: one original 512×512 chest radiograph and four sets of wavelet detail maps. After concatenation, these inputs form a single 13-channel tensor.

The encoder consists of residual blocks that combine multiple 3×3 convolutions with ReLU activation and a 1×1 convolution shortcut. This residual strategy helps preserve low-level features and stabilizes training. Each residual block is followed by 2×2 max pooling, progressively reducing spatial resolution while increasing feature abstraction.

At the bottleneck, a residual block with 1,024 filters processes the most abstract features. The decoder then reconstructs spatial resolution using upsampling layers paired with attention gates for each skip connection. Decoder blocks merge the upsampled features with the gated encoder outputs.

Finally, a 1×1 convolution with sigmoid activation produces the single-channel denoised radiograph, designed to approximate the original full-dose image while retaining clinically relevant details.

0.3 Loss Function

A composite multi-term loss function was designed to balance pixel fidelity, perceptual similarity, edge sharpness, and frequency-domain detail. The following components were used.

0.3.1 Mean Squared Error (MSE)

Pixel-wise fidelity was enforced using the MSE loss:

$$\mathcal{L}_{MSE}(y, \hat{y}) = \frac{1}{HW} \sum_{i=1}^{H} \sum_{j=1}^{W} \big(y(i,j) - \hat{y}(i,j)\big)^2, \quad (1)$$

where y is the ground truth, $\hat{y}$ the prediction, and H, W the image dimensions.

0.3.2 Structural Similarity Index Measure (SSIM) Loss

Structural fidelity was preserved using SSIM[3], which evaluates local luminance, contrast, and structural similarity:

$$\text{SSIM}(x, y) = \frac{(2\mu_x\mu_y + C_1)(2\sigma_{xy} + C_2)}{(\mu_x^2 + \mu_y^2 + C_1)(\sigma_x^2 + \sigma_y^2 + C_2)}, \quad (2)$$

with μ_x, μ_y local means, σ_x^2, σ_y^2 variances, and σ_{xy} covariance. Constants C_1, C_2 prevent division by zero. SSIM was converted to a loss:

$$\mathcal{L}_{SSIM}(y, \hat{y}) = 1 - \text{mean}\big(\text{SSIM}(y, \hat{y})\big). \quad (3)$$

0.3.3 Laplacian Loss

To emphasize edge preservation, Laplacian loss[4] was applied. With L denoting the 2D Laplacian filter:

$$L = \begin{bmatrix} 0 & 1 & 0 \\ 1 & -4 & 1 \\ 0 & 1 & 0 \end{bmatrix},$$

the filtered ground truth and prediction are

$$y^L = y * L, \quad \hat{y}^L = \hat{y} * L, \tag{4}$$

and the Laplacian loss is

$$\mathcal{L}_{Lap}(y, \hat{y}) = \text{mean}\Big(|y^L - \hat{y}^L|\Big). \tag{5}$$

0.3.4 Wavelet Loss

To penalize discrepancies in the frequency domain, wavelet loss was computed by decomposing y and $\hat{y}$ into detail coefficients using multiple wavelet families (`db2`, `coif1`, `sym4`, `haar`). With horizontal (LH), vertical (HL), and diagonal (HH) coefficients:

$$\begin{aligned}\ell_{\text{wavelet}} &= \text{mean}|y^{LH} - \hat{y}^{LH}| \\ &+ \text{mean}|y^{HL} - \hat{y}^{HL}| \\ &+ \text{mean}|y^{HH} - \hat{y}^{HH}|,\end{aligned} \tag{6}$$

encouraging preservation of fine textures and edges.

0.3.5 Composite WEU-Loss

The overall objective combined the four terms:

$$\begin{aligned}\mathcal{L}_{WEU}(y, \hat{y}) = \alpha\,\mathcal{L}_{MSE} + \beta\,\mathcal{L}_{SSIM} \\ + \gamma\,\mathcal{L}_{Lap} + \delta\,\ell_{\text{wavelet}},\end{aligned} \tag{7}$$

with empirically chosen weights $\alpha = 0.1$, $\beta = 0.1$, $\gamma = 0.4$, $\delta = 0.4$. This balanced strong noise suppression with structural detail preservation.

0.4 Qualitative Evaluation Platform

The blinded radiologist assessment (5-point Likert-scale quality rating and image-type classification: original / noisy / denoised) was performed using a custom web-based platform. The evaluation interface and dataset are available at:

`https://smartangio.com/denoisevision/`

References

[1] Shui-Hua Wang, Yu-Dong Zhang, Zhengchao Dong, Preetha Phillips, Shui-Hua Wang, Yu-Dong Zhang, Zhengchao Dong, and Preetha Phillips. Wavelet families and variants. *Pathological brain detection*, pages 85–104, 2018.

[2] Olaf Ronneberger, Philipp Fischer, and Thomas Brox. U-net: Convolutional networks for biomedical image segmentation. In *Medical image computing and computer-assisted intervention–MICCAI 2015: 18th international conference, Munich, Germany, October 5-9, 2015, proceedings, part III 18*, pages 234–241. Springer, 2015.

[3] Zhou Wang, Alan C Bovik, Hamid R Sheikh, and Eero P Simoncelli. Image quality assessment: from error visibility to structural similarity. *IEEE transactions on image processing*, 13(4):600–612, 2004.

[4] Shaohua Li, Xinxing Xu, Liqiang Nie, and Tat-Seng Chua. Laplacian-steered neural style transfer. In *Proceedings of the 25th ACM international conference on Multimedia*, pages 1716–1724, 2017.
